# Inhibition of Transglutaminase 2 by a Selective Small Molecule Inhibitor Reduces Fibrosis and Improves Pulmonary Function in a Bleomycin Mouse Model

**DOI:** 10.3390/cells14070497

**Published:** 2025-03-26

**Authors:** Zhuo Wang, Sriniwas Sriram, Cynthia Ugwoke, Zoe Gale, Maral Tabrizi, Martin Griffin

**Affiliations:** 1 School of Biosciences, College of Health and Life Sciences, Aston Triangle, Aston University, Birmingham B4 7ET, UKz.gale@aston.ac.uk (Z.G.);; 2 Isterian Biotech, Inc., 228 Park Ave S, #66643, New York, NY 10003, USA

**Keywords:** Transglutaminase 2 (TG2), pulmonary fibrosis, idiopathic pulmonary fibrosis (IPF), small molecule inhibitor, transforming growth factor β1 (TGFβ1), α smooth muscle actin (αSMA)

## Abstract

This paper investigates the ability of our selective small molecule TG2 inhibitor 1-155 in reducing fibrosis in a bleomycin-induced pulmonary fibrosis mouse model. Formulated as a fine stable suspension, 1-155 was delivered intranasally (IN) at 3 mg/kg via IN delivery once daily. It significantly inhibited collagen deposition in the lungs in the bleomycin-challenged mice. Compared to its vehicle control treatment, a significant reduction in a key myofibroblast marker α smooth muscle actin and TG2 was also detected in the 1-155-treated animals. Most importantly, 1-155 treatment significantly improved several key lung function parameters, such as cord compliance, vital capacity, and dynamic compliance, which are comparable to that found for the positive control nintedanib at a much higher dosage of 60 mg/kg twice daily via oral delivery. The 1-155-treated mice showed a trend in improvement of average body weight. For the first time, our study demonstrates the effectiveness of a selective small molecule TG2 inhibitor in reducing pulmonary fibrosis in a pre-clinical model. Importantly, we were able to correlate this effect of 1-155 with the improvement of animal lung function showing the potential of the use of TG2 inhibitors as a therapeutic treatment for fibrotic lung conditions like IPF.

## 1. Introduction

Idiopathic pulmonary fibrosis (IPF) is a progressive, irreversible, and fatal lung disease that is characterized by thick collagen and ‘fibroblast foci’ in the lung interstitium, resulting in severe restriction of lung capacity and function [1]. IPF is a rare disease that affects between 200,000 and 300,000 people in the Western world [2]. Current treatments are limited by their lack of efficacy, side effects, or inability to effectively block fibrosis, making IPF a significant unmet medical need.

Research over the past two decades has elucidated several pathological processes that are integral to IPF initiation, including alveolar epithelial cell attrition and activation of transforming growth factor β1 (TGFβ1). However, the molecular drivers of disease progression remain less well defined. Many factors, including association with known environmental agents, connective tissue diseases, and exposure to drugs or radiation therapy have been proposed [3,4], while aging remains the strongest risk factor [5,6].

Transglutaminase 2 (TG2) is a calcium-activated multifunctional enzyme well known for its ability to form protein crosslinks through ε-(γ-glutamyl) lysine isopeptide bonds, which leads to resistance to proteolytic cleavage and, ultimately, stabilization and increased stiffness of the extracellular matrix (ECM) [7]. The role of TG2 in human disease has been widely reported, such as in fibrosis [8,9,10] and cancer [11]. During fibrosis, TG2 was shown to be involved in the accumulation of the large latent TGFβ1 complex into the ECM which facilitates release of the latent TGFβ1 activation protein LAP from the large latent TGFβ1 complex. Active TGFβ1 from LAP promotes fibroblast-to-myofibroblast transition [8,9], Epithelial–Mesenchymal Transition (EMT) [12], and Endothelial–Mesenchymal Transition (EndMT) [13] and increased expression of TG2 in M2 macrophages. This induces ECM protein expression and deposition while inhibiting matrix degradation pathways, leading to matrix accumulation and progressive fibrosis. Importantly, TGFβ1 increases the expression of TG2, forming a self-propagating cycle [11].

The importance of TG2 in IPF is well established, with high levels of TG2 detected in IPF patient lung tissues [9] while TG2 knockout mice develop significantly less pulmonary fibrosis compared to wild type mice when treated with bleomycin [14].

In our previous paper, we demonstrated the presence of increased TG2 in IPF lung fibroblasts and demonstrated a relationship between TG2 and TGFβ1 in IPF fibroblasts dependent on TG2’s crosslinking activity [9]. Importantly, we showed the potential of using site-directed TG2-selective inhibitors as therapeutic agents for IPF by demonstrating that our TG2 selective inhibitor 1-155 can reduce matrix deposition and reverse the myofibroblast phenotype in IPF cells via preventing the activation of TGFβ1. This reduced myofibroblast biomarkers, such as α smooth muscle actin (αSMA), and inhibited excessive matrix protein deposition [9]. Importantly, 1-155 treatment led to the reduction in TG2 expression and ECM deposition in both primary human IPF and TGFβ1-treated human pulmonary fibroblasts, indicating the potential of TG2 as a disease target for IPF [9].

In other studies, we demonstrated that treatment with the TG2-selective and irreversible inhibitor 1-155 resulted in up to a 40% reduction in collagen deposition in a mouse Angiotensin II model of nephrosclerosis and a 60% reduction in infarct size in an acute myocardial infarction mouse model [8]. Earlier studies with our peptidic inhibitor R281 reduced collagen deposition in a rat diabetic nephropathy model [15]. These studies, together with the data from TG2 knockout mice, support the potential use of site-directed TG2-selective inhibitors in the treatment of lung fibrosis.

Here, for the first time, we demonstrate that our TG2 inhibitor 1-155 reduces fibrosis in vivo using a bleomycin mouse model of lung fibrosis with 1-155 administered via the intranasal route. Importantly, we show that 1-155 improves mouse lung function, correlating with its effect on inhibiting fibrosis.

## 2. Materials and Methods

Reagents and antibodies. The general reagents were purchased from Sigma-Aldrich (Dorset, UK), unless stated below.

Antibodies used in this study, including a rabbit monoclonal antibody against αSMA (EPR5386), a rabbit polyclonal antibody against TG2 (AB421), and a rabbit IgG control monoclonal antibody (EPR25A), were purchased from AbCam (Cambridge, UK). The secondary antibody was supplied in the DAB kit. TG2 inhibitor 1-155 (with >95% purity) was synthesized by Wuxi AppTec (Shanghai, China).

### 2.1. Cell Culture

Human pulmonary lung fibroblasts (PromoCell, Heidelberg, Germany) were cultured in fibroblast culture medium containing 2% (*v*/*v*) fatal calf serum, 1 ng/mL human fibroblast growth factor, and 5 µg/mL insulin (PromoCell, Heidelberg, Germany).

### 2.2. FN Staining

FN deposited by primary lung fibroblasts was detected as described previously [16]. The cells (7 × 10^4^/chamber in complete medium) were cultured for 72 h in chamber slides in the presence of TG2 inhibitor 1-155 (between 25 and 500 nM), while DMSO was used as the vehicle control. The cells were cultured with anti-FN antibody for 2 h and then fixed, permeabilized, and incubated with FITC-conjugated secondary antibody for 2 h at 37 °C. The cells were finally mounted with Vecta shield mounting medium (Vector Laboratories, Peterborough, UK) and the fluorescence signals visualized using a Leica epi-fluorescent microscope (Milton Keynes, UK). The fluorescence signal was measured using ImageJ (version 1.53e) and the cell EC_50_ was analyzed using GraphPad Prism (version 9.4.1).

### 2.3. Compound Formulation

The physical and thermal properties of compound 1-155 as its native material (powder form) and formulated as a suspension were characterized by Polarized Light Microscopy (PLM) (LV100PL, Nikon, Nishioi, Japan). The native 1-155 powder was in its crystal form with a particle size of around 4–5.0 µm as measured by PLM. The particles were milled to achieve a smaller particle size of around 2–3 µm. A formulation of the 1-155 milled form was made using 0.1% (*v*/*v*) Tween 80 in 0.9% sodium chloride (*w*/*v*), pH 7.13, which formed a homogenous stable suspension at 1 mg/mL and 2.5 mg/mL with particle sizes in the respiratory range. The 1-155 native powder form was made in 5% DMSO + 95% (*v*/*v*) sulfobutylether -beta-cyclodextrin (SBECD), pH 7.7, which contains 20% SBECD in water (*w*/*w*). Formulations were prepared fresh daily prior to administration and kept at 5 °C.

The formulation of nintedanib was prepared in a 0.1% hydroxyethyl cellulose to a final concentration of 6 mg/mL.

### 2.4. General Animal Study Design

All animal-related research was conducted by CROs in accordance with 2010/63/EU and the national legislation regulating the use of laboratory animals in scientific research and for other purposes. Necessary procedures were taken to ensure randomization and blinding to avoid bias. The tissue samples were coded for histological analysis. The animals were acclimatized for at least a week prior to the start of the experiments.

### 2.5. Lung PK Analysis of 1-155

Lung PK study was conducted by Wuxi AppTec. Animal facilities and Animal Care and Use Program (Units 001369 & SYXKS(沪)2019-0024) are fully accredited by AAALAC (Association for Assessment and Accreditation of Laboratory Animal Care, International) and approved by the Science and Technology Commission of Shanghai Municipal Government. The AAALAC Guideline and the Chinese National Standards related to the care and use of laboratory animals were followed. In vivo protocols were approved by the institutional ethical committee IACUC (Institutional Animal Care and Use Committee), all in-life procedures were performed by trained and experienced scientists, in full compliance with the relevant international standards, EST123, Directive 2012/63/EU, OLAW PHS (Policy on Humane Care and Use of Laboratory Animals).

C57BL/6 male mice (body weight of 25 g, GemPharmatech, Nanjing, China) were used in the studies. The mice were kept in GC type IVC mouse cages with corncob bedding and maintained under standard laboratory conditions (temperature 20–26 °C, relative humidity 30–70%, 15~20 air changes per hour, artificial lighting with circadian cycle of 12 h). Pelleted food and water were provided ad libitum.

The study was divided into three groups and dosed with 1-155, 1 mg/mL with no suspension, 1 mg/mL with suspension, and 2.5 mg/mL of 1-155 with suspension. Compound 1-155 was given through intratracheal (IT) administration in a volume of 30 uL/mouse. At each time point, the animals were euthanized by CO_2_ and 400 μL blood was collected from three mice in each group. Then, the animal was perfused with ice cold 0.9% saline by systemic circulation. BALF samples were collected from the lung, then lung tissue was separated carefully and snap frozen into liquid nitrogen. The blood samples were centrifuged at 1560 g for 10 min at 4 °C to yield around 150 μL plasma. The plasma samples, lung tissues, and BALF samples were stored under −80 °C until LC-MS/MS analysis was performed. Plasma, BALF, and lung tissue samples were collected at 10 min, 30 min, 1 h, 2 h, 4 h, 6 h, and 24 h and analyzed for 1-155 using HPLC-MS using a UPLC (Shimadzu) chromatographic system equipped with an AB Sciex QTRAP 6500 mass spectrometer operated by Analyst 1.6 software packages (Applied Biosystems). Chromatographic separation was carried out through a Waters XBridge BEH C18 column 50 × 2.1 mm ID, 2.5 µm at a flow rate of 0.7 mL/min at room temperature. All data were acquired using Analyst 1.6 software (Applied Biosystems). Plasma concentration versus time data were analyzed by non-compartmental approaches using the WinNonlin software program (version 6.1, Pharsight, Mountain View, CA, USA). Non-compartmental analysis (Phoenix WinNonlin 8.3) was used to determine PK parameters.

### 2.6. In Vivo Efficacy Study Protocol

The efficacy was conducted by Selvita S.A. All animal-related research was conducted in accordance with 2010/63/EU and the national legislation regulating the use of laboratory animals in scientific research and for other purposes (Official Gazette 55/13). An Institutional Committee on Animal Research Ethics (CARE-Zg) oversees that animal-related procedures are not compromising animal welfare.

Additionally, 12-week-old C57BL/6 male mice (Charles River, Italy) were used in the studies. The mice were kept in type III polysulfonate cages with ALPHA-dri dust free bedding and cotton nestles and maintained under standard laboratory conditions (temperature 22± 2 °C, relative humidity 60 ± 5%, 15 air changes per hour, artificial lighting with circadian cycle of 12 h). Pelleted food and water were provided ad libitum.

Lung fibrosis was induced by intranasal (IN) administration of bleomycin (Santa Cruz Technologies, Dallas, TX, USA) with 30 µg/50 µL of 0.9% saline per mouse, which is approximately 1 mg/kg. Seven days after the bleomycin challenge, the animals were randomly divided into different treatment groups. Prior to IN administration, the mice were anesthetized with ketamine +xylazine combination. The control group received saline alone. Table 1 shows the efficacy study design.

The animals were examined clinically twice daily and monitored according to the clinical signs and parameters based on a humane endpoints scale. The animals were weighed on D0, D3, and D6. D7 animals were weighed every day until D21.

On day 21, the mice were anesthetized and lung function measurements were performed, while the rest of the animals were overdosed with ketamine hydrochloride (Narkamon) and xylazine (Xylazine) administered intraperitoneally. In addition, the lungs of all animals were removed, weighed, formalin-fixed, and finally paraffin-embedded for histopathological analysis and analyzed together as one group.

Treatment design is shown in the table below.

### 2.7. Lung Function Measurement

Lung function measurements were performed on 8 animals/group (apart from 6 animals for the saline control and 5 animals for 1-155 IN treatment). The tests performed included Boyle’s law functional residual capacity (FRC) tests, quasistatic Pressure Volume tests (PV), and fast flow volume maneuver plus resistance and compliance tests. The mice were anesthetized with ketamine +xylazine combination. A pulmonary function test (PFT) was performed with measurements on Buxco^®^ Pulmonary function testing system (DSI, MN, USA). The animals were intubated extra-orally and placed on a mechanical ventilator. A tracheal tube provided direct access to the lungs. Parameters measured using this technique included pulmonary resistance and dynamic lung compliance.

In the Buxco^®^ software (version 2.9.0), a new PFT study was created, and animals were allocated to new measurement groups. The mice were anesthetized for surgery and after approx. 10 min, the mice were tracheotomized. Tracheostomy was performed by cutting skin in the neck area and releasing the trachea from the surrounding tissue. Tracheal tubes were placed into the trachea and fixed with a tied suture. Standard 18-gauge stainless steel tubes were used for mice and shortened to 25 mm length. The mice were then loaded into a plethysmograph for testing. Three semiautomatic maneuvers were performed: Boyle’s law functional residual capacity (FRC), quasistatic Pressure Volume test (PV), and fast flow volume maneuver.

Data were calculated using automated data acquisition software Buxco^®^ FinePointe™. Statistical significance was determined from mean values using *t*-test (significance level set to 0.05).

### 2.8. Histopathological Evaluation

Whole lungs were embedded in paraffin and stained according to Crossman‘s Trichrome method. Pulmonary histological changes were assessed using Matsuse modification of the Ashcroft score. Slices of the whole pulmonary area were subjected to histopathological analysis and evaluated at 10× magnification and a total Ashcroft score for each animal was calculated as a mean value.

Statistical analysis and graphical presentation were performed using GraphPad Prism software (version 9.4.1). Outliers were identified using Grubbs test. For the evaluation of the Ashcroft score results, non-parametric statistics (Wilcoxon Signed Rank Test, Mann–Whitney test) was performed using group median. Differences between groups were considered statistically significant when *p* < 0.05.

### 2.9. Histological Analysis of Lung Tissues

Histochemistry for collagen analysis was performed using Picro-Sirius red staining. Immunohistochemistry was performed to detect the presence of TG2 and αSMA in the lung tissue slides. Following xylene dewaxing, the tissues were rehydrated using a gradient of alcohol (100–70%) and antigen retrieval was performed in a citrate-based antigen retrieval solution (pH 6) at 100 °C. Following rinsing the samples, the samples were blocked with 5% BSA in PBS, pH 7.4, for 1 h at room temperature. Novolink DAB kit (Leica) was used to block the endogenous hydrogen peroxidase. Suitable primary antibody in 1% BSA in PBS, pH 7.4, was incubated with the tissues at 4 °C overnight. Following rinsing, the slides were incubated with Novolink polymer for 30 min. The slides were rinsed, and signals were revealed using a DAB Substrate Buffer before drying and mounting the slides using Vector mounting media. Images were obtained using an EVOS imaging system.

Image analysis was conducted using Image J software. Data are expressed as the mean ± SEM for at least three independent replicate experiments. Statistical analysis was undertaken using Microsoft Excel, one way ANOVA with Bonferroni correction post hoc test, and a *p* value of <0.05 was considered to indicate statistical significance. Subsequent F-test two samples for variances and identification of significant differences between individual groups by Student’s *t*-test or Welch’s *t*-test were carried out.

### 2.10. Tolerability Study Setup

The tolerability study was conducted by Selvita S.A. See Section 2.6 for further information.

The mice were assigned to 1 control group and 2 testing groups, each consisting of 6 males per group. Compound 1-155 at a dose of 3 mg/kg and 10 mg/kg was administered IN once daily for 14 days, while Group 1 received the vehicle (0.1% Tween 80 in saline (0.9% sodium chloride) alone as a control.

Subsequent observations and examinations included clinical signs (daily), body weight (daily), food consumption (daily), clinical laboratory investigation, and bioanalysis evaluation. At termination, following macroscopic examination, organ weights were determined and histopathology performed for selected tissue (lung and trachea).

## 3. Results

### 3.1. Optimization of 1-155 to Mouse Lung

**Figure 1:**
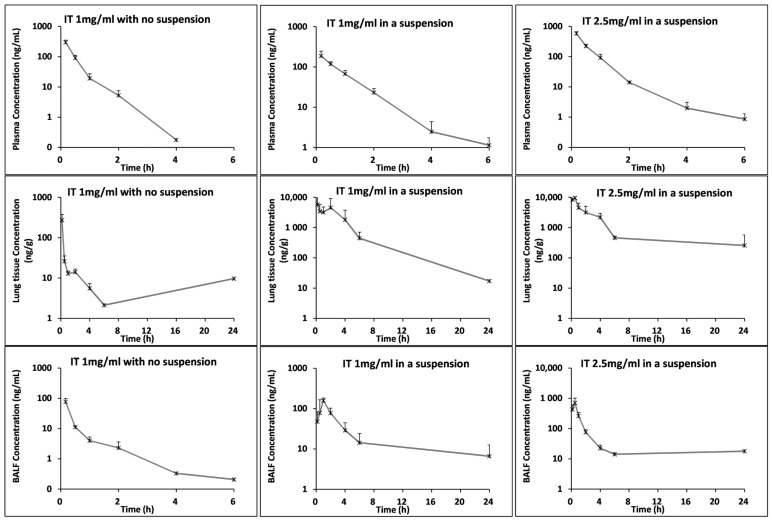
Graphical representation of concentration of 1-155 in lung tissue, BALF and plasma over time using different doses and vehicles where 1-155 concentration in each lung compartment is measured in ng/mL or ng/g for lung tissue.

When delivered to the mouse lung via the intratracheal (IT) route, compound 1-155 (1 mg/kg) as a solution is rapidly absorbed through the lung into the plasma over the first two hours (Figure 1). When delivered as a 1 mg/kg stable suspension via IT, 1-155 showed increased retention in the lung tissue and in the bronchial alveolar lavage (BALF) (Figure 1) when compared to the native 1-155 non-suspension. When using a 2.5 mg/kg IT dosing in suspension, similar or higher sustained exposure in the BALF and lung tissue was achieved in comparison to both the 1 mg/kg suspension and the non-suspension delivered 1-155 (Figure 1).

### 3.2. Effects of 1-155 on Lung Fibroblasts

**Figure 2:**
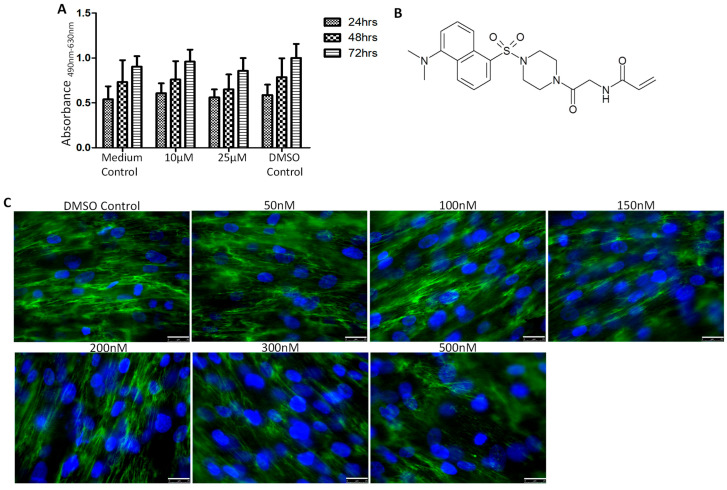
Effects of 1-155 on viability and FN deposition by pHPFs. (**A**) pHPFs (5000 cells/well in 96-well plates) were treated with 1-155 at 10 µM and 25 µM, while pulmonary cell culture medium and DMSO (1:1000 dilution) were used as control treatments. Cell viability was measured using XTT assay at 24, 48, and 72 h. Absorbance at 490 nm and 630 nm was measured using plate reader. n = 5. (**B**) Structure of 1-155. (**C**) FN matrix deposition by human pulmonary fibroblasts were detected via immunofluorescence staining as described in Materials and Methods. pHPFs were treated with 1 ng/mL of TGFβ1 in presence of TG2 inhibitor 1-155 at concentrations between 50 and 500 nM, while DMSO (1:1000 dilution) was used as vehicle control. Mouse anti-human FN primary antibody and GFP-tagged secondary antibody were used and fluorescence signal was measured using EPI-fluorescence microscope. ImageJ was used to measure fluorescence signal (n = 3). Bar, 25 µm.

To test the toxicity of 1-155 in primary human pulmonary fibroblasts (pHPFs) prior to its use in vivo, the effect of 1-155 on pHPF viability was determined. The cells were treated with 1-155 (at concentrations of 10 µM and 25 µM in DMSO), while DMSO was used as the vehicle control treatment. No inhibitory effect on cell viability was found in the primary lung fibroblasts up to 72 h (Figure 2A).

Cell EC_50_ for 1-155 was measured via investigating its effect on inhibiting TGFβ1-stimulated fibronectin (FN) deposition by primary human pulmonary fibroblasts. Figure 2B shows the structure of 1-155, while Figure 2C shows the extracellular FN via fluorescence staining. The fluorescent signal was used to calculate the cell EC_50_ of 1-155 at 284 nM ± 10 nM.

Given the observation that the presence of 1-155 is maintained in the bronchial alveolar fluid and in the lung tissue for up to 24 h when used at 2.5 mg/kg (Figure 1), 1-155 at this dose was used in future efficacy studies. IN dosing was chosen over IT administration to test the efficacy of 1-155 in the bleomycin model. Dosing was given once a day. Reference to the literature suggests that over time, IN gives more reliable results due to less variation in the data [17].

### 3.3. Measurement of Body Weight in 1-155-Treated Animals and Comparison to Nintedanib and Their Respective Controls

**Figure 3:**
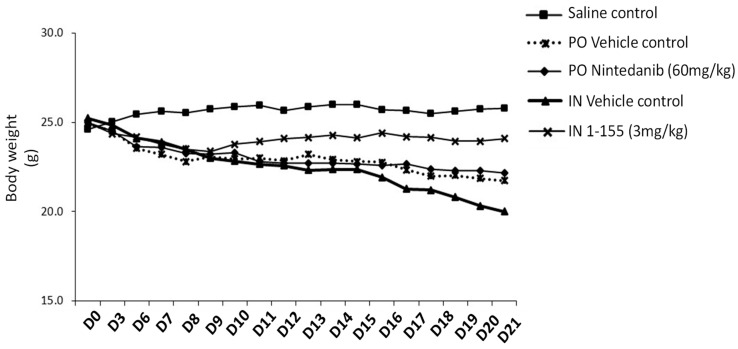
Effects of 1-155 on animal body weight. Measurement of animal body weight was performed every day throughout course of in vivo stage of bleomycin-induced pulmonary fibrosis experiment as described in Materials and Methods. Figure shows average body weight for each group.

To assess the efficacy of 1-155 in inhibiting pulmonary fibrosis, we performed an in vivo study using a bleomycin-induced pulmonary fibrosis mouse model. Throughout the efficacy study, animal body weight was regularly measured for over 21 days. During this period, a significant (*p* > 0.05) difference in the average absolute body weight is shown between all the bleomycin-challenged animals when compared with the saline control between day 6 to day 21 of the experiment. When compared to its corresponding vehicle control, compound 1-155 showed a trend but not significant improvement in average body weight increase from Day 9, which remained stable until D21 when compared to its vehicle control (Figure 3). In contrast, a small but not significant reduction in average body weight loss was observed in the other treated animals, including nintadanib and its vehicle control throughout the course of the experiment (Figure 3).

Low survival rates were observed in all groups treated with bleomycin, compared to the saline vehicle control treatment. A low survival rate (7/16 animals) was observed in the 1-155-treated group (3 mg/kg IN), compared to its vehicle control (7/10 animals).

### 3.4. Histological and Pathological Effects of IN Dosing of TG2 Inhibitor 1-155 on Lung Fibrosis in Bleomycin Mouse Model

**Figure 4:**
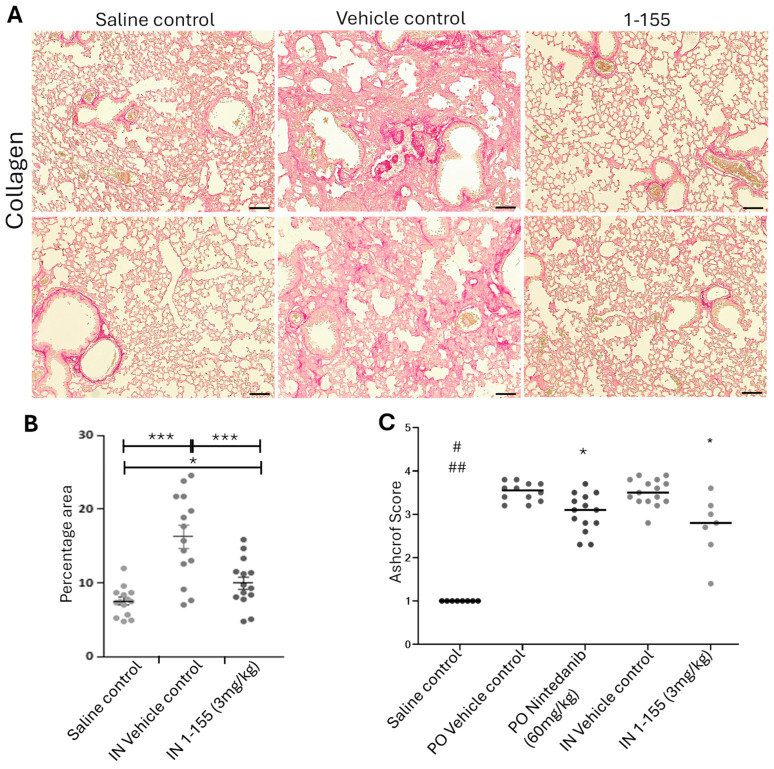

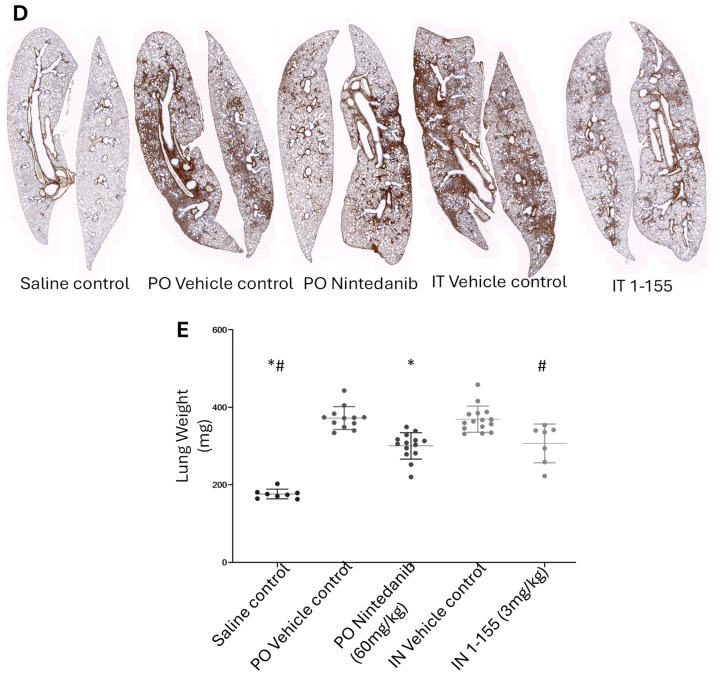
Effects of 1-155 on pulmonary fibrosis in bleomycin mice model. (**A**,**B**) 1-155 significantly reduced collagen deposition in lung tissues compared to saline and vehicle control groups. (**A**) Representative images of collagen via Picro-Sirus Red staining. Staining was performed as described in Materials and Methods. Images were captured using an EVOS imaging system and ImageJ was used for image analysis as shown in (**B**). *, *p* < 0.05; ***, *p* < 0.0001 n = 7, 2 tissues/animal. Bar, 100 µm. (**C**,**D**) Ashcroft analysis of fibrosis shows reduction in lung fibrosis in 1-155-treated animals following bleomycin treatment. (**C**) Ashcroft scores based on level of fibrosis in lung specimen. *, *p* < 0.05 against corresponding vehicle control; #, *p* < 0.05 against PO vehicle control; and ##, *p* < 0.05 against IN vehicle control. (**D**) Representative images of collagen staining using anti-collagen 1A antibody via immunohistology analysis. (**E**) Lung weight. Total weight of lung tissues was analyzed. *, *p* < 0.05 against corresponding vehicle control; and #, *p* < 0.05 against PO vehicle control.

In the bleomycin-challenged groups, increased collagen was found using Picro-Sirius red staining in the lung tissues when compared to the non-bleomycin saline controls. This increase in collagen levels was significantly reduced in the 1-155-treated animals (Figure 4A,B). Ashcroft score analysis of the lung tissues was carried out after Crossman’s Trichrome staining to further quantify the presence of collagen in lung slices (Figure 4C,D). As shown in Figure 4C, the fibrosis observed was patchy in some animals but in others, it had become more established and spread across the lungs. Bleomycin treatment in the vehicle control-treated groups scored over 3.5 by the Ashcroft score. Treatment with 1-155 at a dose of 3 mg/kg IN significantly reduced collagen deposition when compared to the corresponding 1-155 vehicle group (Figure 4A,B). Using the Ashcroft score, nintedanib (60 mg/kg/PO/BID), the positive control treatment, also showed significantly reduced collagen deposition when compared to the bleomycin-challenged group administered with its vehicle control PO BID. However, 1-155 showed a lower average Ashcroft score of 2.7, compared to the nintedanib group of 3.0 (Figure 4C). In both the nintedanib and 1-155 groups, the lung weights were significantly reduced compared to their respective vehicle controls (Figure 4E).

### 3.5. Effects of 1-155 on Inhibiting Myofibroblast Transition Induced by Bleomycin Treatment

**Figure 5:**
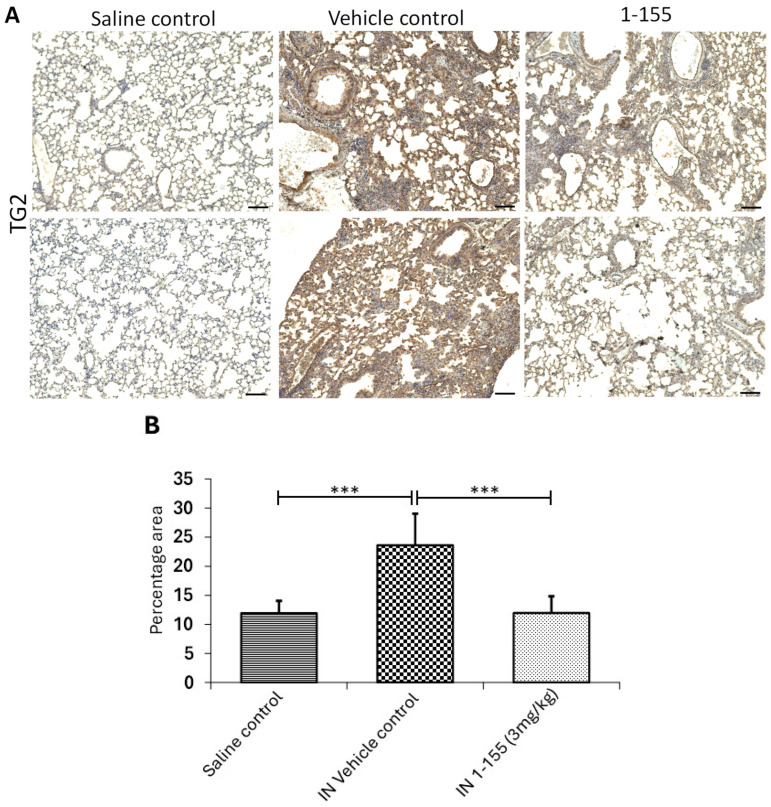

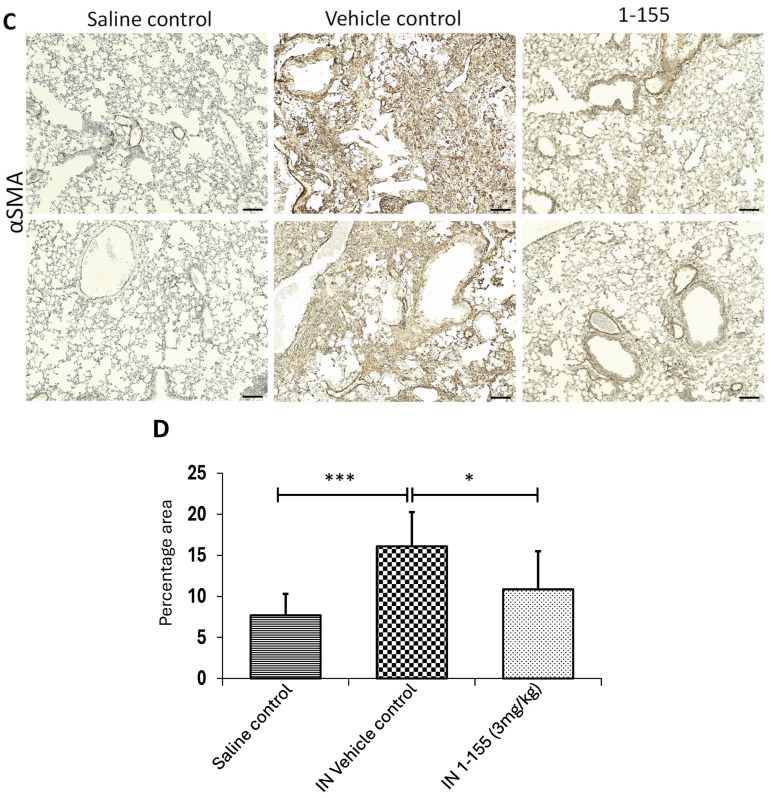
The effects of 1-155 on the presence of TG2 and αSMA antigen in the lung tissues in the bleomycin mouse model. As described in Materials and Methods, a rabbit anti-TG2 polyclonal antibody and a rabbit anti- α-SMA polyclonal antibody were used to detect TG2 (**A**,**B**) and α-SMA (**C**,**D**) in the lung tissues in the bleomycin-induced pulmonary fibrosis mouse model via immunohistochemistry. The samples from the saline control, 1-155 IN, and its vehicle control treatments were analyzed. Representative images of TG2 (**A**) and α-SMA staining (**C**) are shown. ImageJ was used for data analysis, as shown in (**B**,**D**). *, *p* < 0.05; ***, *p* < 0.0001. n = 6. Bar, 100 µm.

The increase in myofibroblasts transitioned from pulmonary fibroblasts in the lungs is one of the major causes for the increase in fibrotic collagen deposition. Therefore, it is important to investigate the mechanism of reduction in collagen deposition in the 1-155-treated animals. Immunohistology revealed a significant increase in TG2 compared to the lungs of the bleomycin-treated animals dosed with 1-155 vehicle control and the saline control. A significant reduction in TG2 was found in the 1-155-treated groups (Figure 5A,B). Importantly, immunohistology also revealed a significant reduction in the myofibroblast marker αSMA in the lungs of the animals treated with 1-155 compared to the vehicle control treatment (Figure 5C,D), indicating a reduction in myofibroblasts in the lungs of the 1-155-treated group. The density of staining for αSMA was lower than that for TG2, which might be expected given that TG2 is found in both the extracellular and intracellular space.

### 3.6. Effect of 1-155 on Pulmonary Function in Bleomycin Mice Model

**Figure 6:**
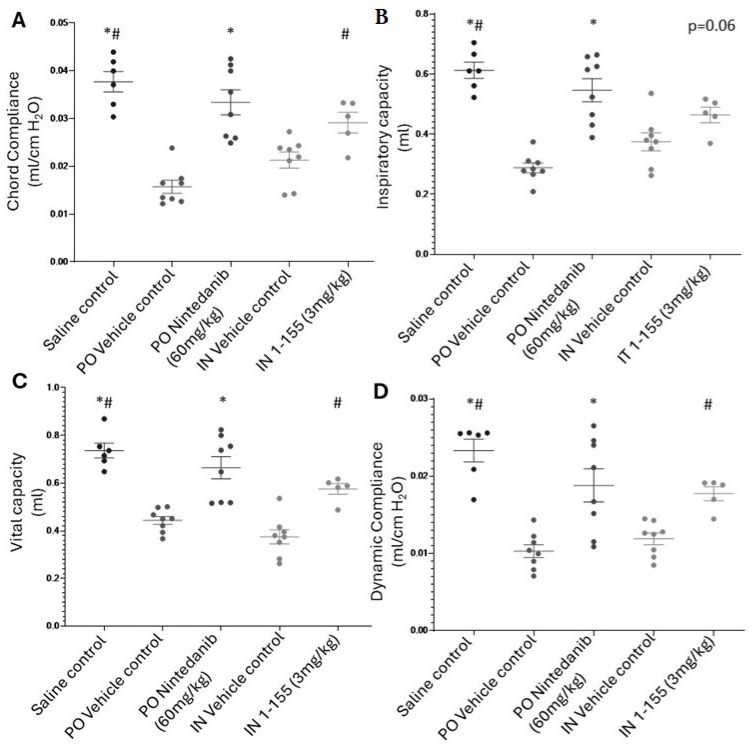

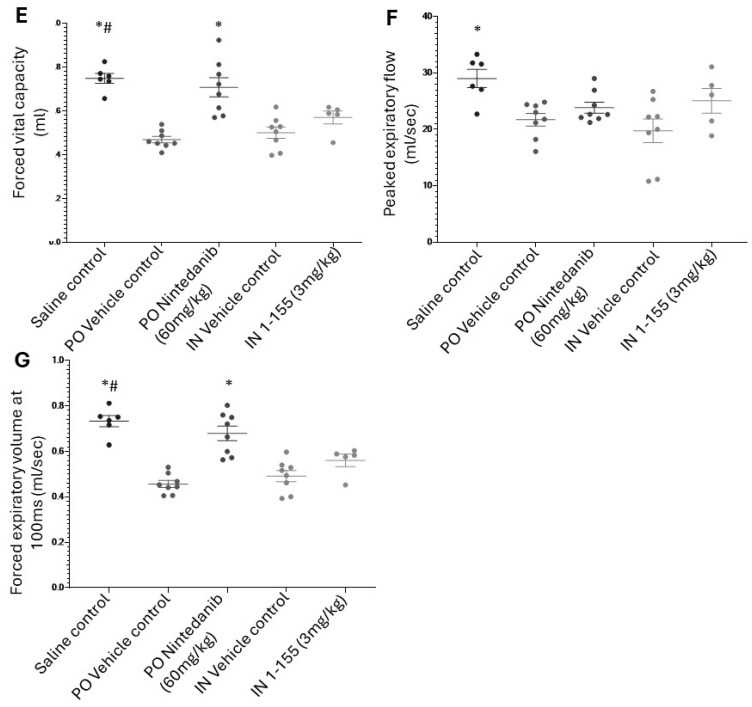
Effects of 1-155 on pulmonary function and lung weight. Pulmonary fibrosis was induced by administration of bleomycin via IN as described in Materials and Methods, while saline was used as control treatment for bleomycin. 1-155 was administrated at 3 mg/kg via IN daily, positive control treatment nintedanib was administrated orally at 60 mg/kg twice daily, while individual vehicle controls for these treatments were used. Lung function tests were performed prior to culling, as described in Materials and Methods. Lung function tests include chord compliance (**A**), inspiratory capacity (**B**), vital capacity (**C**), dynamic compliance (**D**), forced vital capacity (**E**), peaked expiratory flow (**F**), and forced expiratory volume at 100 ms (**G**). *, *p* < 0.05 vs. PO vehicle control. #, *p* < 0.05 vs. IN vehicle control.

The development of fibrosis induced by bleomycin led to a decline in pulmonary function in both bleomycin-challenged vehicle control groups when compared to the saline control (Figure 6A–G). This included effects on total lung capacity, dynamic compliance, chord compliance parameter (as a downward and right-shifted P-V curve), inspiratory capacity and vital capacity, forced vital capacity, forced expiratory volume at 100 ms (FEV100), and peak expiratory flow.

The bleomycin-challenged mice treated with 1-155 IN at 3 mg/kg, when compared to its corresponding vehicle control, exhibited a significant (*p* < 0.05) improvement in lung function parameters, including chord compliance (Figure 6A), vital capacity (Figure 6C), and dynamic compliance (Figure 6D), while improvement of inspiratory capacity was also observed with *p* = 0.06 (Figure 6B). In addition, even though not statistically significant when compared to its vehicle control group, 1-155 improved the lung function parameters of forced vital capacity, peak expiratory flow, and forced expiratory volume at 100 ms (Figure 6E–G). At a much higher dose of 60 mg/kg via oral administration twice daily, improvement of lung functions in the positive control nintedanib was observed (Figure 6A–E), when compared to its vehicle control.

### 3.7. Tolerability Study of 1-155 in Healthy Animals

To test the tolerability in vivo of the 1-155 treatment used in the bleomycin-induced pulmonary fibrosis efficacy study, a tolerability study was undertaken using healthy animals where 3 mg/kg and 10 mg/kg of 1-155 in suspension was administered IN once daily for 14 days. No mortality or clinical signs of toxicity occurred during the study. Importantly, there were no effects on body weight or food consumption and no microscopic findings were observed during the treatment period with either dose.

Histopathological evaluation did not reveal any abnormalities for the trachea when compared to the controls. For the different lung lobes observed, the lobes showed a slight increase in (sub-) acute alveolar/interstitial inflammation when compared to the controls, which was not considered serious. No obvious signs of toxicity were observed in the vehicle control.

## 4. Discussion

Several studies have identified TG2 as a promising disease target for IPF [9,14,18]. It was reported that when challenged with bleomycin, TG2-deficient mice showed less fibrosis and metabolic changes in the lungs, compared to the wild type animals [19]. However, the use of small molecule selective TG2 inhibitors, according to the available literature, has not been tested for efficacy in animal lung fibrosis models. Current IPF treatments, nintedanib and pirfenidone, can slow the rate of decline in lung function and disease progression. Nintedanib is a pan intracellular inhibitor, targeting multiple growth factors like tyrosine kinase receptors, including PDGF receptors, VEGF receptors, and FGF receptors [20]. Pirfenidone functions via affecting various pathways, including interfering with growth factors (e.g., TGFβ, PDGF, and basic FGF), upregulating MMPs to attenuate matrix accumulation, modulating cytokines (e.g., interleukins), and regulating the activity and proliferation of T and B lymphocytes [21]. Given the broad spectrum of physiological and pathological pathways affected by these treatments, they can cause side effects, such as diarrhea and nausea. Cases of abnormal liver function in nintedanib patients were reported, and both drugs are not recommended for patients with severe liver disease [22,23]. Therefore, there is an urgent unmet clinical need for novel treatments that are specific to fibrosis.

Our previous work has demonstrated that there is a self-propagating cycle between TG2 and TGFβ1 in driving fibroblasts into myofibroblast transition during IPF, which can be inhibited by TG2 small molecule inhibitor 1-155 [9]. 1-155 is a TG2 inhibitor with high potency and selectivity, and has been effective in inhibiting fibrosis in several in vivo fibrosis models, including acute myocardial infarction [8] angiotensin II-induced cardiac and renal fibrosis [14]. No toxic effects were observed in these animal models for over 21 days when 1-155 was delivered s.c. using a mini pump [8,10].

Our current challenge in developing 1-155 as a treatment for fibrotic disease is its relatively high clearance, which may potentially limit systemic side effects but would limit the concentration distribution into the lungs when administrated IV or orally [10]. Therefore, we have overcome these characteristics of 1-155 by administering it to the lung directly via the IN route. The advantage of topical administration via inhalation of the drug as used in humans should improve its potency and once cleared rapidly in the plasma, limit potential side effects.

By delivering 1-155 in a stable suspension IN, we demonstrate that 1-155, which has good cell permeability [10], has a better chance to be retained by permeating into the lung sub-compartments and providing longer duration in the lung. At 3 mg/kg in suspension, 1-155 treatment resulted in a significant reduction in fibrosis when the presence of collagen in the lung tissue was analyzed in both lung tissue sections and the whole lung slices. Compared to its vehicle control, 1-155 treatment significantly reduced the Ashcroft scores, an indicator of pulmonary fibrosis, with a reduction in lung weight. Importantly, 1-155 showed a lower average Ashcroft score (2.7) than the positive control treatment nintedanib (3.0). Our findings agree with the effect of 1-155 on inhibiting fibrosis in the heart and kidneys, as we reported previously [8,10].

Further analysis revealed that the inhibition of bleomycin-induced pulmonary fibrosis was in part due to a significant reduction in lung myofibroblasts, as confirmed by the myofibroblast marker αSMA. Furthermore, 1-155 treatment led to the reduction in the high level of TG2 in bleomycin-treated lungs, validating that TG2 could be a reliable disease marker for progressive pulmonary fibrosis [9,14], such as IPF. In the future, it would also be interesting to explore the effect of 1-155 on pulmonary fibrosis in a longer-term chronic fibrosis model and investigate the possibility of co-staining intracellular TG2 with αSMA positive cells [9].

The overall aim of our study was to demonstrate the potential of 1-155 as a treatment for IPF. Therefore, it was important for us to correlate pathology data with clinical readouts, i.e., the lung function analysis. For the first time, we are able to demonstrate a very clear correlation between the inhibition of pulmonary fibrosis by TG2 inhibitor 1-155 both by histopathology and its effects on improving lung function using the bleomycin-induced pulmonary fibrosis mouse model. Significant improvements in key lung function parameters by 1-155 include cord and dynamic compliance. This suggests that 1-155 treatment can help alleviate the restrictive problems associated with a diseased lung, as seen in the bleomycin-treated lungs of mice and in patients with IPF where myofibroblast proliferation and increased deposition of collagen restricts access of air to the lung tissue. In line with the improvements shown in compliance, an improvement was also shown for lung vital capacity and inspiratory capacity (*p* = 0.06), suggesting these parameters were improved with 1-155 treatment. Although not significant, forced vital capacity and peaked respiratory flow were also improved by 1-155 treatment, compared to its vehicle control. Forced vital capacity decline is predictive of mortality in patients with IPF and is presently used as a clinical trial endpoint to define disease progression [24]. It is important to note that a much higher dosage of nintedanib (60 mg/kg, twice daily) was used compared to 1-155 (3 mg/kg, twice daily).

In a tolerability study, 1-155 delivered IN at 3 mg/kg and 10 mg/kg in healthy animals caused no serious toxic effects over 14 days and in the efficacy study, 1-155-treated animals did show a small improvement in body weight between day 9 to day 21. However, despite the above observations, a lower survival rate was observed in the animals treated IN with 1-155 when bleomycin treatment was introduced.

## 5. Conclusions

Our work demonstrates, for the first time, that a TG2 selective small molecule inhibitor can inhibit pulmonary fibrosis and improve lung function in an in vivo bleomycin-induced pulmonary fibrosis mouse model. We also demonstrate a correlation between the in vivo effect of TG2 inhibition by 1-155 and the myofibroblast-mediated pulmonary fibrosis and further confirmed TG2 as a therapeutic target for pulmonary fibrosis. Importantly, we have shown the potential of a highly potent and selective TG2 inhibitor as an inhaled therapeutic for lung fibrosis such as IPF, opening up opportunities for future exploration of these inhibitors as topical treatments for pulmonary fibrotic diseases like IPF.

## Figures and Tables

**Table 1:** Efficacy study treatment design.

Group	Treatment	Animal Number
Saline control	Saline IN, once daily	8
Vehicle control for nintedanib	0.1% hydroxyethyl cellulose (HEC) PO, twice daily	16
Reference treatment nintedanib	60 mg/kg in 0.1% hydroxyethyl cellulose (HEC) PO, twice daily	16
Vehicle control for 1-155	0.1% Tween 80 in 0.9% NaCl IN, once daily	10
Experimental treatment 1-155	3 mg/kg in 0.1% Tween 80 in 0.9% NaCl IN, once daily	16

## Data Availability

The original contributions presented in this study are included in the article. Further inquiries can be directed to the corresponding authors.
